# Magnetic Assisted Navigation in Electrophysiology and Cardiac Resynchronisation: A Review

**Published:** 2006-10-01

**Authors:** AS Thornton, M Maximo Rivero-Ayerza, LJ Jordaens

**Affiliations:** Clinical Electrophysiology, Thoraxcentre, Erasmus MC, Rotterdam, The Netherlands

**Keywords:** Arrhythmias, catheter ablation, radiofrequency energy, magnetic navigation, cardiac resynchronisation therapy

## Abstract

Magnetic assisted navigation is a new innovation that may prove useful in catheter ablation of cardiac arrhythmias and cardiac resynchronization therapy. The ability to steer extremely floppy catheters and guidewires may allow for these to be positioned safely in previously inaccessible areas of the heart. The integration of other new technology, such as image integration and electroanatomic mapping systems, should advance our abilities further. Although studies have shown the technology to be feasible, with the advantage to the physician of decreased radiation exposure, studies need to be performed to show additional benefit over standard techniques.

## Introduction

### Where are we now in catheter ablation and resynchronisation therapy?

Catheter based ablation of cardiac arrhythmias has advanced significantly since its inception, with progress both in the technology, as well as in our understanding of both simple and complex arrhythmias. Newer approaches to ablation such as in atrial fibrillation and ventricular tachycardia, as well as more defined endpoints for these procedures, has meant that success rates, both acutely as well as in the long term, have improved. Success rates for less complex procedures such as AVNRT, accessory pathways and atrial flutter ablation are above 95%, while those for more complex intraatrial reentry, ventricular tachycardia and atrial fibrillation are, in the best hands, around 80%.

Notwithstanding these excellent results there is still a not insubstantial failure rate. In addition, in certain anatomies, such as repaired complex congenital heart disease, ablation of some arrhythmias can be extremely challenging. Besides the intellectual challenge these arrhythmias may pose, there is still a marked technical challenge in manipulating conventional catheters to sites which need to be reached. Today's catheters, while a significant advance on prior generations, are still fairly stiff, as they use pull-wire technology to bend and straighten the catheter in a fixed curve, and require twisting of the catheter to rotate the tip. This stiffness decreases their manoeuvrability in certain situations and increases the risk of perforation when compared to softer catheters. New developments such as electroanatomical mapping systems mean that even more technology needs to be incorporated into the design of modern ablation catheters.

A recent innovation in heart failure therapy is that of cardiac resynchronisation therapy (CRT), where, in addition to the standard pacemaker or ICD leads, an additional lead is used to pace the left ventricle. In most cases the left ventricular lead is placed via the coronary sinus (CS) and its branches so as to pace the epicardial surface of the left ventricle. In approximately 90% of patients this is successfully achieved, but there may be a number of impediments to success. The anatomy of the CS and its tributaries is extremely variable, in normal individuals, and even more so in patients with underlying cardiac disease. Entering the CS os, and then manipulating the lead into the targeted side branch can be time consuming and in some cases impossible due to tortuousity or angulation of the CS or its side branches. Even if one is able to access the targeted side branch there may a high threshold or phrenic nerve stimulation. This may then require repositioning of the lead into another side branch. In CRT we have been forced to use some of the skills of our interventional colleagues to improve our success rates, most particularly by learning to manipulate coronary guidewires in order to be able to access some of the coronary veins. The CS differs significantly in its diameter from the coronary arteries and thus the use of guidewires is more difficult. Invariably after one has used a curve to engage a particular side branch, a different curve to the tip of the guidewire might be useful to advance the guidewire further. Using the "normal" guidewire this is not possible without removing and reshaping it. This may either not be possible or extremely time-consuming. Technology has developed significantly since the inception of this therapy, with the development of better leads and improved delivery systems. The search continues however for further improvement in success rates, as well as for a decrease in procedure, and associated fluoroscopy time.

Of course, whether it is a catheter ablation or CRT, the operator is standing, or perhaps sitting next to the patient, wearing a lead apron, but still being exposed to radiation. The risk from radiation imposed by newer X-ray systems is significantly less than with older systems, but we should still strive for less, both for the patients and of course, with the cumulative exposure, for ourselves. The stress imposed on the musculoskeletal system, particularly to the lumbar spine, by the standing and the weight of the lead apron has caused a premature end to some careers.

### What alternatives would we like to have?

This is by no means an exhaustive list, but amongst those to be considered are:

- More steerable and softer catheters which could be manipulated to previously inaccessible or markedly problematic areas while improving safety.
- Catheters which could be manipulated with better precision so as to improve the quality of arrhythmia mapping.
- Coupling these desired alternatives with the latest technology, such as image integration and electroanatomic mapping, to increase success rates in the therapy of increasingly complex arrhythmia substrates.
- New guidewire technology to allow access to difficult CS anatomy and allow delivery of pacing leads to these areas.
- A system which would reduce the X-ray exposure of the operator and would improve operator comfort and safety. A system which would allow us to perform the procedure remotely from a comfortable chair in the control room would be ideal.

### What is magnetic assisted navigation (MAI)?

One of the options which may provide a solution to some of the concerns and give us some of the alternatives discussed above is magnetic assisted navigation. A system has been developed which uses a powerful external magnetic field to orientate tiny magnets contained in the tip of extremely flexible catheters or guidewires with the large external magnetic field. Orientating these tiny magnets points the tip of the catheter or guidewire to where you want it to go, and by using an advancer/retractor system the catheter or guidewire can then be manoeuvred around inside a cardiac chamber or a vessel, such as the coronary sinus or a coronary artery. It is important to emphasise that the external magnetic field does not pull or push the tiny magnets and the catheters or guidewires in which they are contained. By understanding the physical characteristics of the catheter or guidewire, and how it moves in the heart or vessel, software can be developed to allow the cardiologist to direct the movement of the tip of the catheter or guidewire to access the region of interest.

### Magnetic assisted navigation - the system

**Figure 2:**
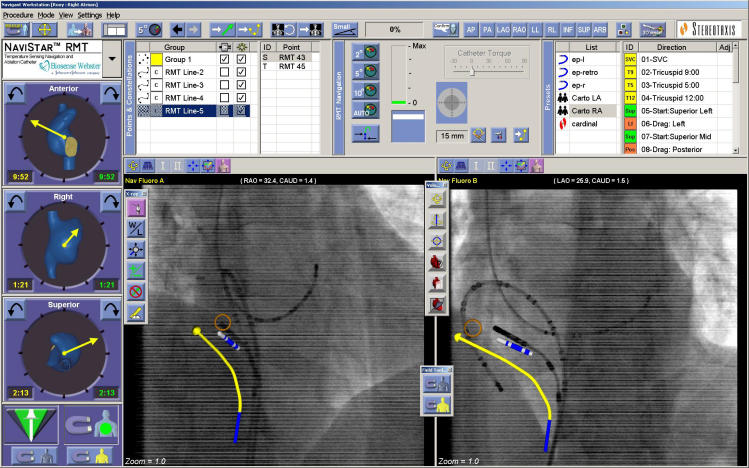
The Navigant screen. The fluoroscopic images at the bottom (RAO on the left and LAO on the right) can be stored at the start of the procedure, but in this case have been recently updated. The yellow line is the virtual catheter which the software has placed on the image. The blue and white catheter tip is the virtual catheter which the CARTO system places in the Navigant screen. As can be seen the virtual catheter is overlying the actual fluoroscopic catheter shadow. The other features on the screen are aids to navigation.

**Figure 3:**
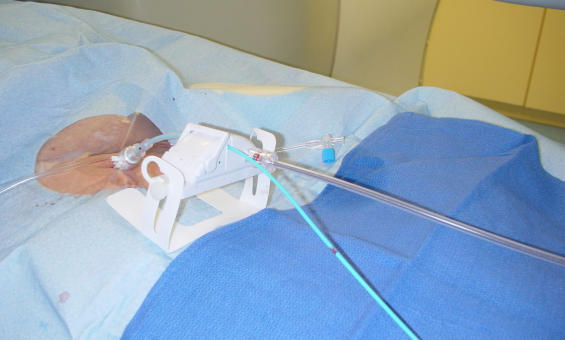
The catheter advancer system. The catheter runs through this simple mechanical cogwheel system and advances or retracts depending on the direction of movement of the cogwheel. An adapter connects the catheter advancer to the sheath to prevent prolapse of the catheter between the two.

**Figure 4:**
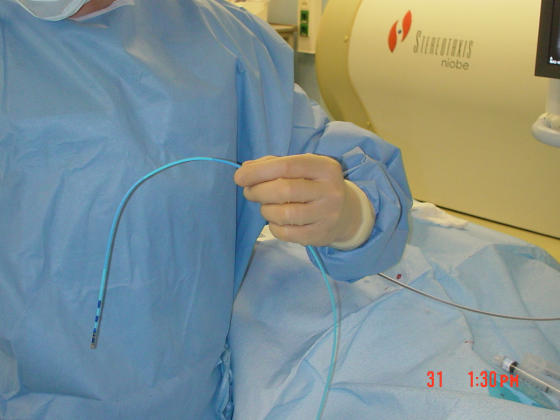
A magnetic catheter outside the magnetic field. It is extremely flexible. There are magnets in the tip and underneath the two blue markers on the distal shaft just proximal to the tip.

**Figure 5:**
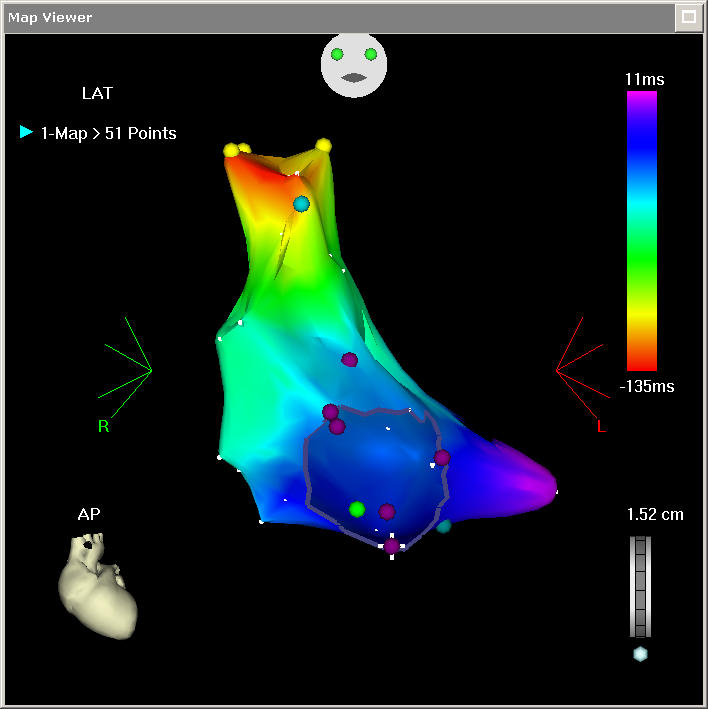
CARTO RMT - a 51 point map which reflects the right atrial anatomy reasonably well. This map was made in 10 minutes and shows a focal SVC tachycardia.

**Figure 6:**
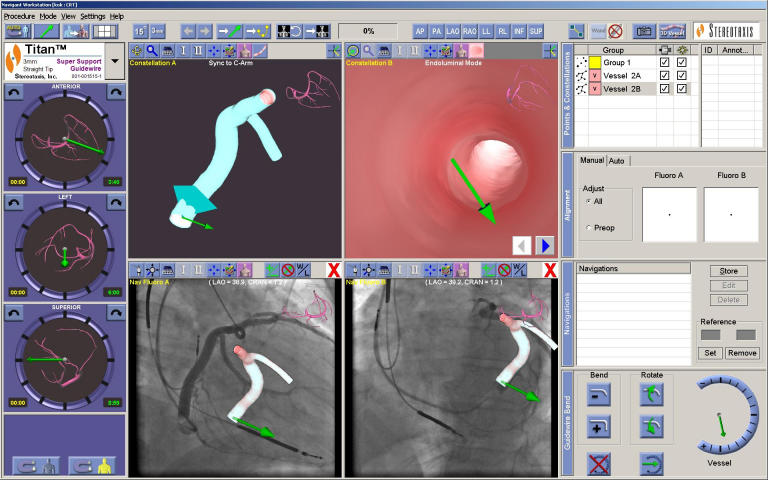
PAIEON - This image again shows the Navigant screen with the reconstruction overlaid on the radiographic images saved during the angiogram. The image above the LAO radiographic image shows the 3D reconstruction and the image above the RAO shows a fly-through image from the reconstruction. This is then used to navigate the magnetically enabled guidewire into the CS side branch. The other features which can be seen are aids to navigation.

The basic system developed by Stereotaxis (St Louis, MO, USA) for magnetic assisted navigation (MAI) consists of a number of different parts:

1. The Niobe system (Stereotaxis) consists of 2 large permanent external neodymium-iron-boron magnets located on both sides of the patient table (Figure 1a and 1b). In the Niobe 1 iteration these can only be swung in (active navigation) or stowed, while in the Niobe II the magnets have a different housing and can also be tilted to allow for more angulation of the single plane C-arm imaging system. The magnetic field generated by interaction of these two magnets forms a uniform field of 0.08 tesla (T) within a spherical volume with a diameter of 15 to 20cm; sufficient to encompass the heart when the patient is properly positioned.
2. The Navigant system (Stereotaxis) is the computerised graphical user interface system and includes the software used for image integration, and for control of the magnetic fields that orientate the catheter within the heart (Figure 2). Software has been developed which, by understanding the characteristics of the catheter or guidewire and how it moves in the heart or vessel, allows the cardiologist to direct the movement of the tip of the catheter or guidewire to access the region of interest. The operator can access an area of interest using either vector based navigation or target based navigation. In vector based navigation the operator tells the system, by drawing a vector in virtual 3D space on the computer, what orientation of the magnetic field he requires. In target based navigation a target is placed on a specified point using the stored orthogonal fluoroscopic views, and the system then calculates the vector required to access this target. Each time a vector is selected or a target is marked, the computer sends information to the magnets which changes their relative orientation, and with it the orientation of the uniform magnetic field in the chest, so that catheter orientation is then changed within a matter of a few seconds. The software contains a number of preset vectors selected by the manufacturer, after careful appraisal of multiple CT images and reconstructions, for positioning the catheter at various anatomic landmarks. In addition the software can be used to automatically map various chambers of the heart.
3. Once the catheter is orientated it may need to be advanced or withdrawn in order to approximate the area necessary. This is done using a simple mechanical advancer, the catheter advancer system (Stereotaxis) (Figure 3). At present a commercially available advancer system is only available for use with ablation catheters. Guidewires need to be advanced and retracted by hand, although we and others have modified the present system to also be able to advance and retract guidewires remotely. A commercial advancer system for guidewires is in development.
4. The next parts of the system, and of great importance, and the reason for all the above complex technology, are the electrophysiology ablation catheters and the guidewires. These are extremely flexible distally, especially the distal shaft of the ablation catheters, and have tiny magnets (single or multiple in various configurations) inserted in their distal portion (Figure 4). The catheters and guidewires are made either by Stereotaxis or partner companies.
  The latest catheters have 3 tiny magnets distributed along the distal shaft and the tip of the catheter to increase responsiveness of the catheter to the magnetic field generated. The maximum tissue force which can be applied by one of these flexible catheters is less than the average, and significantly less than the maximum which can be applied using a standard catheter.1 It would appear almost impossible to perforate a vessel or chamber with one of these catheters, and this has not been described to date. At present only a 4mm tip radiofrequency ablation catheter is available, but irrigated RF, and 8mm tip RF catheters are due for release within the next year. Already available are catheters which can be used with an electroanatomical mapping system (CARTO, Biosense Webster, Diamond Bar, CA, USA) - see below.
  The initial guidewires available were essentially standard floppy PTCA guidewires to which a magnet had been attached to the end and although usable, were not ideal. The newest generation of guidewires have been better designed to work with the system and retain a fairly floppy tip with a magnet, but have significantly more support - very useful when trying to track an LV lead over the wire into a CS side branch.
5. Of course the system is combined with an X-ray system - mainly a monoplane unit because of the limitations imposed by the magnets, although a biplane system can be installed for use when the magnets are stowed and not in use. Because of the magnets, the rotation of the imaging system is limited to approximately 30 degrees RAO and LAO in Niobe I and nearly 45 degrees with Niobe II.
6. Finally, an electroanatomic mapping system and image integration have also been integrated with the system both for ablation, and , in the case of image integration, for coronary artery and coronary sinus interventions.
  - In the case of ablation this is with the CARTO system - now known as CARTO RMT (Figure 5), which has been specifically redesigned to work in the magnetic environment of Stereotaxis. CARTO RMT includes all the latest updates such as CARTO Merge, where a 3D reconstruction of a CT or MRI can be integrated into the electro-anatomic map. With the CARTO integration there is communication between the two systems allowing for real time catheter orientation and positioning data to be sent from CARTO to the Stereotaxis system, and for the catheter tip to be displayed on the saved images stored on the Navigant system. This permits tracking of the ablation catheter without having to update the radiographic image as often. Magnetic vectors can also be applied from the CARTO screen. A feature called design line can be used to send a line of points - either for mapping a specific area, or potentially as a line of ablation points. "Click and go" is a utility allowing for one to click on an area of the map to set a target and have the system guide the catheter to this point. As Stereotaxis and CARTO have feedback integration, the CARTO system is able to feed back to the Stereotaxis system if the exact point is not reached, allowing for further automatic compensation by the software until the desired point is reached.
  - In the case of CRT or interventional coronary work PAIEON (Paieon, Haifa, Israel) (Figure 6) is a system which allows one to make a 3D reconstruction of a vessel from 2 or more radiographic views. Once this has been done virtual fly-though views can be generated which, after registration of the catheter tip on the image, assist in navigation of the catheter up to and into a side branch os.

### What does it offer us?

All the components of the MAI as well as the X-ray, ablator, and stimulator can be operated from the control room. Therefore, after initial placement of sheaths and catheters, the entire procedure, in the case of ablation, can be performed remotely from the control room. This eliminates the exposure to radiation for the physician and also decreases the strain from standing next to the bed for long periods wearing a lead apron. In the case of CRT the benefit of remote navigation is less as most of the procedure cannot be performed with Stereotaxis and at present the guidewire still needs to be advanced and retracted manually. The latter problem will be solved by the availability of a commercial advancer system.

Using the above components it is possible to move the catheter or guidewire in extremely small increments of 1 degree or 1mm within the heart and vessels, making mapping more accurate. All vectors and targets selected can be saved, as can relative positions of the catheter advancer system, allowing one to reproducibly revisit specific areas in the heart or side branches of vessels. Because of the flexibility of the catheters, perforation is extremely unlikely and has not been described to date.

## Studies on magnetic assisted navigation to date

The studies to date on using MAI in catheter ablation, CRT or coronary intervention have largely been feasibility studies and there have been no large-scale comparisons with manual manipulation of catheters or guidewires. Initial studies have also tended to focus particularly on mapping or ablation of less complex arrhythmias, where the advantages of MAI would be more difficult to demonstrate. Given that a recurring theme in all the papers is that there is a steep learning curve, it would also seem warranted to get over this first before comparing this new technology with established therapies. We will review here the studies published to date as well as discuss some of our own feelings on MAI.

### Catheter ablation

The initial studies in catheter ablation were performed with an older 0,15 T magnet version of the system, initially in animal, and then in human studies [1,2]. These studies were mainly mapping studies, where floppy magnetically enabled catheters were navigated to pre-specified sites with good success, both on the right and left sides of the heart. The electrogram signals obtained were comparable, as were the stimulation thresholds. In addition catheter stability was thought to be good during the cardio-respiratory cycle as well as during tachycardia. In one of these studies 13 patients with SVT were studied [2]. Of these 5 had manifest accessory pathways, right lateral in one and left sided in 4 (3 were mapped transseptally, and 1 retrograde), while 7 were AVNRT and 1 was flutter. 2 of the WPWs and 5 patients with AVNRT underwent successful ablation.

Using MAI for ablation of AVNRT has been shown to be feasible without complications in almost 100 published cases, with similar procedure times and success rates. In those where there was a comparison with standard catheters there was significantly reduced radiation exposure for the physician [3-;5]. Even in the early studies without comparison to standard catheters it immediately became clear that physician fluoroscopy times are significantly reduced as the physician is exposed only during advancement of catheters to the heart. These procedures are already those with fairly short fluoroscopy times, and the added benefit in procedures commonly associated with much longer fluoroscopy times is obvious. Of importance is that even at this early stage of our experience fluoroscopy times for the patients were not prolonged and it can be anticipated that these will shorten even further, especially when the system is used together with non-fluoroscopic electroanatomic mapping systems. Catheter stability, even during junctional rhythms, appears to be excellent. In one of the studies there was a trend to decreased radiofrequency ablation time.5  Slow pathway modification has even been performed using this system in patients with somewhat more challenging anatomy such as with a persistent left sided SVC [6].

Very little has been published to date on ablation of accessory pathways, however some of this data has been presented in abstract form. The usual variety of accessory pathways have been ablated using MAI including anteroseptal accessory pathways [7], with success rates generally as expected from manual catheters. Mapping may in fact be enhanced by the ability to move more precisely around the annulus of either the tricuspid or mitral valve. The ability to return to a given point accurately using stored vectors adds to the ease of mapping, and given the fact that less force is applied with these catheters the risk of mechanical block is probably decreased. Catheter stability has again been seen to be excellent. We have shown that a retrograde approach to left sided accessory pathways is feasible and gives us alternatives when a transseptal approach cannot be used for some reason.

We have published a study on the use of MAI in RVOT VT, as well as a case study where we used the system to ablate an idiopathic left ventricular fascicular VT [8,9]. When performing RVOT VT ablations we found that mapping of the RVOT was markedly facilitated by use of the remote magnetic navigation, enabling very fine movements to be made in order to optimize activation- and pacemapping. In addition, the use of such a floppy catheter avoided the catheter induced extrasystoles that are sometimes troublesome during these procedures. The results are good and again physician fluoroscopy time was markedly reduced. We have not ablated ischaemic VTs with this system, but have used it for the initial mapping, in the RV and LV, both retrograde and transseptal. A retrograde approach to left ventricular VT appears a little more difficult because of catheter stability across the aortic valve, however the transseptal approach has given excellent maps with good resolution.

Greenberg et al first published results of pulmonary vein isolation using the magnetic system in an animal model [10]. They successfully isolated the upper pulmonary veins in 7 dogs without complication or long term stenosis using a transseptal approach.  Because these procedures were performed remotely the radiation exposure of the physician was significantly reduced. They also noted that they had been able to navigate to 30 of 30 pulmonary veins attempted in 5 dogs using a retrograde approach. Pappone et al have described in detail their initial human experience using Stereotaxis together with the CARTO RMT system to perform circumferential pulmonary vein ablation [11]. They found the system very useful for making the map, while acquiring more points in less time than would have been possible doing a manual map. They felt that the initial learning curve was extremely steep but fairly short and that despite longer procedure times in this initial group of patients, that mapping and ablation times were shorter and that they had a similar success rate. Ablation times for the right pulmonary veins particularly were shorter than when using standard catheters. There were no complications. They felt that using the system made the ablation potentially less operator dependent, although this remains to be proven.

Our own experience, part of which is detailed above, is now over a 100 cases, and worldwide is over 2000 cases. The only limiting factor to routinely performing other types of ablations (typical atrial flutter, AF ablation, ischaemic VT, etc) is the lack of availability of irrigated tip, and 8mm tip ablation catheters. As mentioned above, we have also used the system extensively for mapping in combination with CARTO RMT and have found it extremely versatile, with the ability to easily form accurate and detailed maps both in the atria and the ventricles. The possibility of using a retrograde approach for ablation of atrial fibrillation and complex corrected congenital heart disease is being explored.

### Cardiac resynchronization therapy

MAI is increasingly being used for positioning a guidewire in the target CS side branch to aid in placement of the left ventricular lead in pursuit of CRT. In a small published series [12] we showed that using MAI and the first generation of guidewires, procedure and fluoroscopy times were similar, as was success compared to manual placement of the guidewire. Pacing and sensing characteristics did not differ between the two groups. No complications occurred. We have also used MAI for placement of LV leads without the use of a CS sheath [13]. This may be useful in decreasing the hardware use and cost as well as decreasing the risk of perforation of the right side of the heart or coronary sinus dissection. While this approach is feasible, the procedure using the presently available guidewires is longer, with more radiation exposure for the patient, but not the physician. The difficulties with the original guidewires was that positioning in the selected side branch was somewhat complicated by a lack of stiffness of the guide wire with some buckling at the SVC - right atrial junction and within the right atrium. Two further problems with using MAI for CRT are that the magnets are unfortunately situated directly where one would stand and thus cause an impediment, and that at present there is no commercially available guidewire advancer. The latter means that someone has to manually advance and retract the wire to command. The availability of an advancer will significantly improve this situation.

We are presently assessing the utility of the PAIEON system in conjunction with newer wires to improve the success rate of side branch cannulation, with a decrease in fluoroscopy time. A further potential advantage of MAI is that the vectors used to access a particular side branch can be stored thus allowing for investigation of multiple side branches for optimal positioning without extracardiac stimulation, and then a rapid return to the best site. This needs to be confirmed in clinical investigation.

### Coronary intervention

We have not gone into detail here as this is beyond the scope of our interaction with the system. The initial problems we experienced with the guidewires have to some extent been mirrored by the interventional cardiologists, who were able to access difficult anatomy with the guidewire, but were then unable to deliver balloons and stents to this area. Newer wires are overcoming this problem and there are an increasing number of publications regarding the utility of MAI for coronary interventional work [14-17].

## Conclusion

We have, in this review, highlighted the present situation with regard to catheter ablation of cardiac arrhythmias and cardiac resynchronization therapy, and where we might like improvements. We have also provided an overview of what magnetic assisted intervention is and the equipment which is presently available to perform this. In addition, we have reviewed the published literature and hopefully given an insight into the experience with this new technology. It is important to emphasise that the initial enthusiasm for this technology needs to be reinforced now by large trials in experienced centres comparing it to accepted practice.

## Figures and Tables

**Figure 1a:**
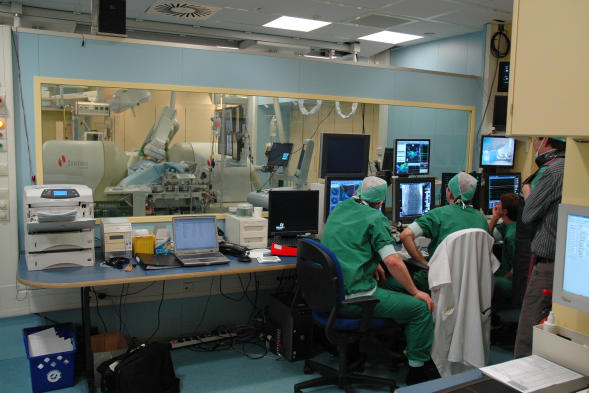
The Stereotaxis room with the Niobe I magnets and single plane C-arm. The limited angulation of the C-arm can be seen. In this case CARTO RMT is being used to map. As can be seen, all aspects of the study are being performed from the control room. There is a nurse maintaining contact with the patient and monitoring the patient's haemodynamics.

**Figure 1b:**
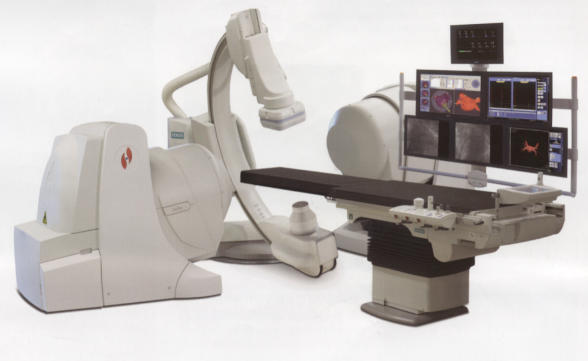
Niobe II with magnets which can be tilted thus increasing the angulation possible with the C-arm.
